# Diagnostic significance of the novel biomarker combination for early-stage non-small cell lung cancer detection: results of the blind clinical study

**DOI:** 10.3389/fonc.2025.1508563

**Published:** 2025-05-19

**Authors:** Janneta Tcherkassova, Evgueni Klinski, Sergey Tsurkan, Anna Prostyakova, Alexander Boroda, Yuliya N. Pirogova, Leonid N. Bagmet, Marina Sekacheva

**Affiliations:** ^1^ Research & Development Department, Universal Cancer Marker (UCM) Technologies, Toronto, ON, Canada; ^2^ Laboratory Polymers for Biology, Shemaykin-Ovchinnikov Institute of Bioorganic Chemistry RAS, Moscow, Russia; ^3^ World-Class Research Center “Digital Biodesign and Personalized Healthcare”, Sechenov First Moscow State Medical University, Moscow, Russia; ^4^ Sechenov First Moscow State Medical University, Sechenov University, Moscow, Russia

**Keywords:** lung cancer, CA-62, tumor markers, CT, pulmonary nodules

## Abstract

**Background:**

NSCLC can be cured in up to 65% of cases if detected early. However, most of the lung cancer (LC) cases are diagnosed at an advanced stage.

**Objective:**

The assessment of various tumor markers in retrospective double-blind clinical study and their possible combinations for detection of early-staged non-small cell lung cancer (NSCLC); evaluation of the best TM panel as a pre-screening tool for LC before Low-Dose CT scan; the development of the protocol for future prospective clinical study.

**Methods:**

A double-blind clinical study was conducted on 304 clinically verified patients, including 141 NSCLC, 133 healthy volunteers and 30 patients with COPD. Quantitative measurement of various TM was carried out using commercial immunoassays.

**Results:**

Unlike other tumor markers, which are expressed proportionally to the tumor growth, CA-62 demonstrated the highest values at Stage I and II of NSCLC. The use of CA-62 for early-staged NSCLC achieves 92% sensitivity at 95% specificity (AUC = 0.973). The diagnostic value of the best TM signature (CA-62, CEA and CYFRA 21-1): 100% Specificity, 90% Sensitivity, and 94% test accuracy, AUC=0.990.

**Conclusions:**

The results of the study demonstrated that the TM combination allows increasing the Specificity for patients with indeterminate pulmonary nodules detected by CT scans and improves the accuracy of differential diagnosis.

## 1 Introduction

Lung cancer (LC) is the leading cause of cancer mortality with the highest cancer prevalence in many countries with developed economies. The high lung cancer age standardized (ASR) mortality rate (18 in 100,000) in the world is related to its high prevalence (11.4%) and the steady increase (about 2,200,000 per year) of diseases affecting the working-age population, as well as to the difficulties of timely diagnosis (1). Lung cancer kills more patients every year than prostate, breast and colon cancer combined (1, 2). Recently Low-dose computed tomography (LDCT) scan of thoracic organs has been growingly accepted as the most effective LC screening method, where the proportion of early stages (I&II) increased from 28.5% to 40% (3). However, in many countries in the world the LDCT method does not allow its wide use within the organized population-based screening due to its limited availability and throughput. Despite encouraging results from several large randomized clinical screening trials involving more than 150,000 people, the medical community is still debating the “cost-effectiveness” of using low-dose CT scans for lung cancer screening due to low population adherence (4, 5).

Barriers to implementing Lung Cancer Screening (LCS) can be categorized into three main groups:

1. Patient Factors that include limited knowledge about Low-Dose Computed Tomography (LDCT) efficacy for cancer screening, some trust issues regarding the healthcare professionals and services; health concerns about radiation exposure from screening procedures; practical obstacles and emotional barriers.
2. Clinical Factors that include: insufficient understanding of LCS guidelines and their benefits, cancer overdiagnosis concerns, time constraints, radiation concerns associated with LDCT, incidental findings of unrelated abnormalities during screening, concerns about patients’ ability to afford the screening, and result follow-up challenges.
3. System Factors that include: insurance coverage for patients, limited availability of CT scanners, difficulty in identifying eligible individuals based on smoking status data, resource imitations related to the Insufficient personnel, equipment, and support services to facilitate screening, and Screening program complexity of implementing comprehensive LCS programs (6).

Screening programs are considered effective according to the World Health Organization (WHO), when they reach over 70% of the at-risk population, provided the required infrastructure and resources are available (7).

It is obvious that early detection of cancer through screening can significantly reduce both morbidity and mortality. At the same time, the ability to simultaneously screen for multiple cancer types offers substantial advantages over single-cancer screening methods. Currently, only five cancers have recommended screening protocols worldwide, and adherence to these guidelines is generally poor to fair, indicating significant potential for improvement (7). Lliquid biopsies also face economic challenges, especially when used for general population screening rather than for targeting high-risk groups. Early-stage cancers often have no symptoms or produce minimal detectable signals, which are precisely the cases where early detection is most impactful (8, 9).

In this context, incorporating additional cancer markers before conducting LDCT scans could address many of the mentioned above barriers to Lung cancer screening, provided the biomarkers are sensitive to early-stage cancers. Considering various limiting factors, a blood draw to obtain samples prior to LDCT is likely to improve patient adherence. Therefore, we pursued a method to increase cancer screening rates through prior biomarker analysis with high sensitivity and specificity. The newly developed cell surface biomarker, CA-62, has demonstrated promising results in detecting precisely early staged cancers, including breast cancer, prostate cancer, colorectal cancer, and lung cancer (10–12). CA-62 specifically targets the N-glycoside portion of peptides unique to poorly differentiated cells, such as embryonic, stem, and cancer cells. Blind clinical studies have demonstrated that elevated CA-62 levels achieve 92% sensitivity at 95% specificity in distinguishing histologically verified cancers from healthy individuals (10–12). CA-62 is a surface biomarker that targets a specific glycosylation pattern characteristic of poorly differentiated epithelial cells. The epitope recognized by CA-62 is not organ-specific but is associated with early oncogenic transformation, where cells begin to lose their normal differentiation patterns and acquire proliferative capacity. This process is common across many epithelial malignancies, including NSCLC. These tumors frequently escape detection in conventional imaging and do not present any clinical symptoms at early stages, making a differentiation-based approach especially relevant.

Since early-stage cancers are characterized by poorly differentiated cells, we also investigated biomarkers that have shown high specificity for lung cancer in previous studies to utilize a combination of serum biomarkers. Depending on the histological classification of LC, it is possible to determine the following biomarkers: neuron specific enolase (NSE) and cancerous-embryonic antigen (CEA) in small cell LC; cytokeratin fragment (CYFRA 21-1), a marker of squamous cell cancer (SCC), and CEA in squamous cell cancer (13); СЕА, CYFRA 21–1 and СА-125 in adenocarcinomas, CYFRA 21-1, SCC and СЕА – in large-cell lung cancer (14, 15). However, the tumor markers (TM) do not have enough sensitivity for detecting early stages of LC that is achievable in combination with highly sensitive and cancer-specific novel biomarker CA-62 (10, 11).

By using a combination of these biomarkers, it is possible to enhance the specificity and sensitivity of lung cancer screening, thereby improving early detection rates and ultimately reducing the burden of lung cancer. Therefore, such combination of biomarkers followed by LDCT potentially can reduce medical costs and can bring regular cancer screening and cancer care to patients. The objectives of the retrospective double-blind clinical study were the assessment of various tumor markers and their possible combinations for detection of early-staged non-small cell lung cancer (NSCLC) and evaluation of the best TM panel as a pre-screening tool for LC before Low-Dose CT scan and the development of the protocol for future prospective clinical study.

In the article we compared the results of the best selected TM panel (CEA, CA-62 and CYFRA 21-1) measurements for 141 NSCLC patients, 30 patients with chronic obstructive lung diseases (COPD) and 133 healthy control subjects. For comparison, serum levels of other well-known TM used in lung cancer diagnosis were measured: CA-125, CA 15-3, CA 19-9, NSE, CEA, and SCC. Quantitative measurement of the biomarkers level was carried out using commercial electrochemiluminescent, chemiluminescent and enzyme-based immunoassays. The measurements were compared with the findings of histopathological diagnosis, which were used as the gold standard for the diagnosis of LC neoplasia, with literature on other LC studies and randomized clinical studies on the use of Low Dose Computed Tomography in lung cancer screening.

## 2 Materials and methods

### 2.1 Clinical study design

A double-blind clinical study using serum samples of patients with histologically confirmed diagnosis of NSCLC was carried out at the First Sechenov University in Moscow, RF. During the clinical study, a group of medical researchers at the Sechenov University did not know the identification of the samples prior to the data analysis. The blinding was performed by third party specialists of the external independent laboratory of the Federal Service for Surveillance for Health Care of the Russian Federation. This research design provides a high level of internal credibility and avoids any bias, randomness or confusion. As a result, the team of independent experts did not produce the identification of the samples until the data had been disclosed.

### 2.2 Ethical approval and informed consent to participate

The study was approved by the local ethics committee of Sechenov First Moscow State Medical University (Sechenov University); protocol №07–17 dated 13.09.2017. The study was conducted in accordance with good clinical practice and the Declaration of Helsinki. All samples from patients included in the study have signed voluntary informed consent to participate in the studies, collecting biological material, clinical data and sharing anonymous data were collected according to approval by the Local Ethics Committee of Sechenov First Moscow State Medical University. A total of 55 patients with histologically confirmed LC and 56 healthy control subjects were included in the analysis. The serum samples were collected at the clinical center of Sechenov University from the study participants after the night fasting and were taken to the clinical laboratory.

### 2.3 Statistical analysis

Statistical analysis of the results, analysis of the ROC-curves, area under curve (AUC) measurement, calculation of the test’s diagnostic characteristics, such as Sensitivity (Sen), Specificity (Sp) and Test Accuracy (Acc) to identify early stages of NSCLC were conducted using the MedCalc Software. The results were considered statistically significant at p< 0.05. The weighted kappa (k) coefficient demonstrate how much one diagnostic test is consistent with another evaluation method taken for true judgment “Gold Standard” (16), was used to further assess the efficacy of the marker application. Kappa coefficients are being interpreted as indices of the test quality in evidence-based medicine. The exact values of the weighed kappa-coefficient demonstrate a significant difference between two diagnostic methods: an agreement between the two estimation methods is considered bad if 0< k< 0.20 and good if k > 0.81.

### 2.4 Serum samples

All serum samples were taken before treatment. This study included 304 blinded by reputable third-party certification company serum samples from 141 histologically verified NSCLC patients with known TNM histopathological classification and staging performed according to AJCC eighth edition (17), 30 patients with COPD and 133 healthy control subjects that have been tested by two independent expert teams in different clinical laboratories. The study included two groups of samples from different sources. Among the 141 NSCLC samples, 44 (31.2%) were at Stage I, 23 (16.8%) at Stage II a-b and 74 (52%) at Stage III a-c. Blind group 1 consisted of 209 serum specimens, including 100 healthy control subjects, Stage I (N=44), Stage II (N=23), and Stage III a-c (N=42) of NSCLC patients. The majority of serum samples in this group (69%) were from patients with early stages of NSCLC (N=67). Serum samples from healthy control subjects (N=100) in group 1, as well as from patients with NSCLC before treatment (N=62) were obtained by venous puncture and were centrifuged and stored at –85°C until assayed according to the standard approved protocol of the First Sechenov University (“Sechenov University”) Moscow, Russia, for prospective research study. Some samples (N=79) from group 1 were obtained from Bio Specimen Bank Precision For Medicine, Inc. (USA). Another blind group 2 of samples consisted of 95 serum samples, including healthy control samples (N= 33), 32 samples from NSCLC patients (IIa-IIIa-c) and 30 samples from patients with COPD was obtained and described by the Institute of Clinical Chemistry (Frankfurt, Germany). Most COPD and NSCLC patients (80%) were chain smokers, with at least 10 years of smoking history. The average age for healthy volunteers was 53 years old (95% CI: 44–85 years), 68 years old (95% CI: 24-93) for NSCLC patients and 66 years old for COPD patients. All patients were Caucasian. Most NSCLC patients (84%) were identified through LDCT screening and the rest 16% were clinically identified. Tumor marker levels were measured on entering the study, without any pre-analytical processing of serum samples. The following variables were assessed in all the patients: age, sex, race, tumor origin, histological type and tumor stage. Quantitative determination of tumor marker levels was determined by commercial kits ELECSYS СА-125, ELECSYS СА 19-9, ELECSYS CYFRА 21-1, ELECSYS SCC (COBAS, Roche Diagnostics GmbH, Germany, EU)] on multifunctional immunoassay analyzer MODULAR ANALYTICS E170 Elecsys (COBAS, Roche Diagnostics GmbH, Germany, EU) in electrochemiluminescence mode. Quantitative measurement of the CEA, CA 15–3 and NSE levels was carried out using ELISA kits CA15-3-IFA-BEST, CEA-IFA-BEST, NSE-IFA-BEST (“VEKTOR-BEST”, Novosibirsk, RF) by the Tecan Spark reader (Germany, EU) using originally collected serum samples. We considered the upper limits of normality to be 5 ng/ml for CEA, 2.5 ng/ml for CYFRA 21-1, 2 ng/ml for SCC, 37 U/ml for CA19.9, 25 ng/ml for NSE, 35 U/ml for CA-125, 30 U/ml for CA 15-3, and 5000 U/ml for CA-62. The serum level of the CA-62 cancer antigen was measured using the chemiluminescent set of reagents CLIA-CA-62 (JVS Diagnostics LLC, RF) on the Tecan Spark reader (Germany, EU) using thermally treated serum aliquots remaining from other cancer marker measurements. All serum samples from groups 1 and 2 were centrifuged (1300 g, 10 minutes) in SST vacutainer tubes with clot activator and separating gel. After that, serum samples were frozen at -86°C before use. Following the measurements for other cancer markers, for CA-62 level’s evaluation all tested serum samples were inactivated by thermal treatment (+56°C, for 30 minutes) using standard protocols for blood draw (18). Serum samples were collected under an IRB-approved protocol from Federal licensed/registered facility following GMPs.

## 3 Results

### 3.1 Descriptive statistics

The diagnostic characteristics of various tumor markers CA-62, CEA, CA 15-3, NSE, SA-125, CA 19-9, CYFRA 21–1 and SCC were compared for 304 serum samples of patients, including 141 NSCLC (Stage I-III), 133 samples from healthy volunteers and 30 samples from COPD patients in a blind clinical study. The majority (54.5%) of NSCLC patients had squamous cell carcinoma, 42% had adenocarcinoma and 3.5% had large cell carcinoma. Almost half (48%) of NSCLC patients had early stages of the disease, while 52% had advanced stages of lung cancer. **Table 1** shows the descriptive statistics of the studied samples for different NSCLC stages, healthy control subjects and patients withCOPD, median and mean (SD, CI 95%) levels of tumor markers CA-62, CEA, CA 15-3, NSE, CA-125, CA-19-9, CYFRA 21-1, and SCC.

**Table 1:** Descriptive statistics of different tumor marker levels in the studied samples.

Tumor marker	Diagnosis	COPD	Healthy control subjects	NCLC All Stages	NSCLC Stage I	NSCLC Stage II	NSCLC Stage III
	Number	30	133	141	44	23	74
CA-62	Mean	3,808	2,652	11,178	12,740	11,261	10,220
	Median	4,016	2,667	10,697	12,822	8,772	10,390
	95% CI	3,410 – 4,206	2,479- 2,824	10,423 – 11,932	11,064 - 14425	8,780- 13,741	9,522 – 10,917
	SD	1,066	1,006	4,531	5,527	5,737	3,011
	Normal distribution	0.55	0.12	0.003	0.21	0.19	0.0001
CEA	Mean	2.4	2.4	17.61	4.484	10.726	27.554
	Median	2.25	2.2	4.6	3.7	5.2	5.1
	95% CI	2.07-2.9	2.2 -2.59	5.8 -29.4	3.56 -5.41	4.36-17.1	5.26 -49.8
	SD	1.1	1.13	70.5	3.04	14.7	96.2
	Normal distribution	0.0006	0.0002	<0.0001	<0.0001	<0.0001	<0.0001
CYFRA 21-1	Mean	2.1	1.609	3.755	1.914	2.801	5.147
	Median	2.05	1.5	2.8	1.95	1.9	4.35
	95% CI	1.82-2.39	1.472-1.746	3.233 - 4.278	1.7666 - 2.1612	1.488 - 4.115	4.364 - 5.929
	SD	0.76	0.79	3.14	0.81	3.04	3.38
	Normal distribution	0.7236	<0.0001	<0.0001	0.0798	<0.0001	<0.0001
NSE	Mean	18.7	12	15.8	8.9	11.61	21.2
	Median	21	12	14	8	11	22
	95% CI	15.8 - 1.5	10.7 - 3.3	13.9 - 7.6	6.7 - 11.2	7.6 -15.6	18.8-23.6
	SD	7.64	7.57	11	7.5	9.3	10.5
	Normal distribution	0.22	<0.0001	<0.0001	<0.0001	<0.0001	0.0009
SCC	Mean	1.317	0.928	2.165	0.98	2.309	2.826
	Median	1.2	1	1.4	0.9	1.1	1.7
	95% CI	1.15 - 1.5	0.8 - 1.0	1.42 - 2.9	0.83 - 1.1	0.22 - 4.8	1.63- 4.0
	SD	0.45	0.49	4.48	0.5	5.85	5.18
	Normal distribution	0.029	0.107	<0.0001	<0.0001	<0.0001	<0.0001

Tumor markers expression levels obtained for CEA (4.2 - 5.1 ng/mL), CA 15-3 (17–27 U/mL), CA-125 (21–28 U/mL), CA 19-9 (12–37 U/ml), NSE (8–22 ng/mL), CYFRA 21-1 (1.9 – 4.35 ng/mL) and SCC (0.9 – 1.7 ng/mL) for NSCLC patients correlated well with literature data for early and advanced stages of non-small cell lung cancer (19–24). Unlike all TM studied in this paper, CA-62 glycoprotein indicated the highest level of expression at Stage I of NSCLC (12,822 U/mL), which remained very high at more advanced stages of cancer: Stage II (8,772 U/mL) and Stage III (10,390 U/mL). Other tumor markers demonstrated lesser sensitivity: CEA (48.2%) > CYFRA 21-1 (44%) > CA-125 (22%) > CA 15-3 (19%) > SCC (18.4%) > CA 19-9 (10%) > NSE (9%).

**Figure 1:**
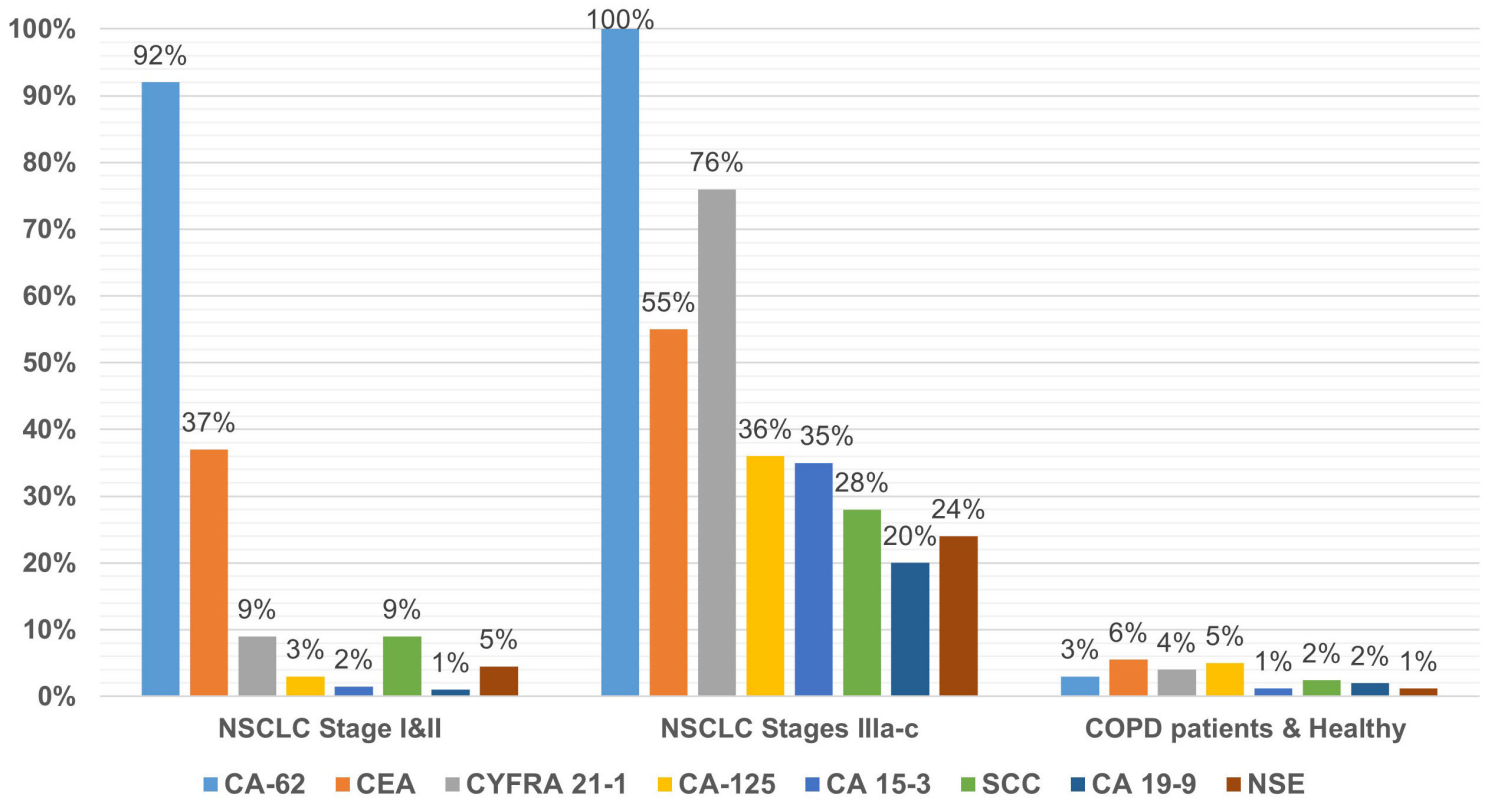
A ratio of patients with elevated level of tumor markers in different cohorts.

Using the standard cutoff values recommended by the commercial test kits manufacturers for ECLIA Elecsys CA-125, CA 19-9, CYFRA 21–1 and SCC, ELISAs CA15-3-IFA-BEST, CEA-IFA-BEST, NSE-IFA-BEST and CLIA-CA-62 were calculated their diagnostic parameters for all NSCLC stages (I-III). The results are presented in **Table 2**. A ratio of serum samples studied with elevated biomarkers within a group of NSCLC patients with early stage (N=67) and advanced stages (N=74), as well as healthy controls subjects and patients with COPD (N=163) are presented in **Figure 1**.

**Table 2:** Diagnostic characteristics of the studied tumor markers for early-staged NSCLC.

Tumor marker	Cutoff value	Sen	Sp	Test accuracy	AUC
CEA	>5 ng/ml	68/141 (48,2%)	153/163 (93,8%)	72,7%	0.852
CA-62	>5000 U/ml	136/141 (96,4%)	158/163 (97%)	96,7%	0.973
СА-62	>5600 U/ml	126/141 (89%)	163/163 (100%)	95,1%	0.973
NSE	> 25ng/ml	13/141 (9,2%)	156/163 (96%)	55,6%	0.600
SCC	> 2 ng/ml	26/141 (18,4%)	160/163 (98%)	61,2%	0.515
CYFRA 21-1	> 2,5 ng/ml	62/141 (44%)	156/163 (96%)	72%	0.598
СА 19-9	> 24 ng/ml	10/141 (7,1%)	155/163 (95%)	54,3%	0.578
CA-125	> 35 U/ml	31/141 (22%)	158/163 (97%)	62,2%	0.511
CA 15-3	> 30 U/ml	27/141 (19%)	161/163 (98%)	61,8%	0.511

A comparison of the studied tumor markers sensitivity of NSCLC early stages (I and II) detection revealed the following pattern: CA-62 (92%) > CEA (37%) > CYFRA 21-1 (9%) and SCC (9%) > NSE (4.5%) > CA-125 (3%) > CA-15-3 (1.5%) > CA-19-9 (1%). The highest sensitivity of NSCLC early stages detection (92%) was observed for CA-62 marker as compared to CEA, CYFRA 21–1 and SCC tumor markers, which are already used in the diagnosis and monitoring of LC (9, 24). At stages III a-c with the lesion of bifurcation lymph nodes or nodules of the mediastinum, the sensitivity of markers is more comparable to the CA-62 cancer antigen and decreases in the following order: CA-62 (100%)< CYFRA 21-1 (75%)< CEA (55%)< CA-125% (36%)< CA 15-3 (35%)< SCC (28%)< NSE (24%)< CA 19-9 (20%).

**Figure 1** shows the percentage of patients (%) in a cohort of early-stage NSCLC (N=67) in a cohort with advanced stages of NSCLC (N=74), in a cohort of healthy control subjects and patients with COPD (N=163). In a cohort of COPD patients, the use of recommended cutoffs for each TM revealed insignificant false positive results ranging from 1.2% for CA 15–3 and NSE markers to 5.5% for CEA (**Figure 1**).

The majority (83%) of patients with COPD had the median concentration of CA-62 carcinoma-specific marker (2,606 U/ml) and CEA (2.3 ng/ml), which were at the same level as healthy controls. Only 3% of the COPD patient’s cohort (4/30) and healthy control subjects (1/133) showed a slight increase in the CA-62 level at the standard cutoff value of 5000 U/ml at 97% Specificity. For carcinoembryonic antigen CEA only 6% of COPD patients demonstrated an elevated marker’s level using a 5 ng/ml cutoff value at 93.8% specificity. Healthy control subjects (N=133) and COPD patients (N=30) showed a normal median expression of CA-62 cancer antigen (2,667 U/mL and 2,664 U/mL) and CEA antigen (2.2 ng/mL and 2.4 ng/mL). We have not found any difference between samples from Bio Specimen Bank Precision For Medicine, Inc. (USA) and samples collected in the hospitals in Russia: the median, an average of each cohort of patients had similar value for samples collected in the USA and in Russia. At the same time, for all other markers medians studied, the medians of healthy volunteers (N=133) and COPD patients (N=30) were in the same range as the early stages of NSCLC (**Table 1**).

The present study was designed as a retrospective double-blind format, which implies a predetermined imbalance in disease prevalence compared to the general population. Specifically, the group of patients with verified diagnosis of cancer was significantly over-represented relative to real-world prevalence, while the control group was designed to be of comparable size to ensure adequate statistical power. While the case-to-control ratio does not reflect population prevalence, it is a standard and appropriate design for estimating the essential diagnostic properties (sensitivity, specificity, AUC) of a biomarker, independent of prevalence. We also note that no statistical feature selection or model-based tuning was applied to optimize the marker combination *post hoc*, which reduces the likelihood of performance inflation due to overfitting.

However, this design does not allow the direct calculation of Positive Predictive Value (PPV) and Negative Predictive Value (NPV), as both are highly dependent on the true prevalence of the disease in the target screening population. Therefore, including PPV and NPV estimates based on the current dataset may therefore mislead readers unfamiliar with the methodological nuances of diagnostic test evaluation. Accurate assessment of these values requires modeling based on population-level prevalence data and would be more appropriate in a prospective study setting.

**Figure 2:**
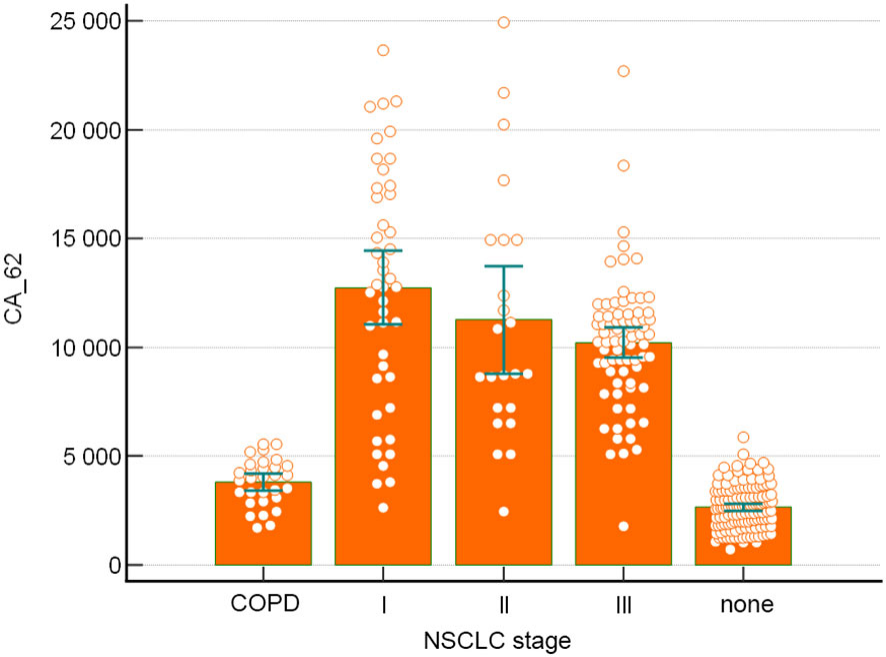
A distribution of clinical samples by NSCLC stage for CA-62 marker.

It is noteworthy to mention that in contrast to the mucins and other tumor markers, which expression is directly proportional to the tumor growth, the marker CA-62 has very high Sensitivity (91-96.4%) at the early stages of NSCLC (**Figure 2**).

### 3.2 ROC curve analysis

**Figure 3:**
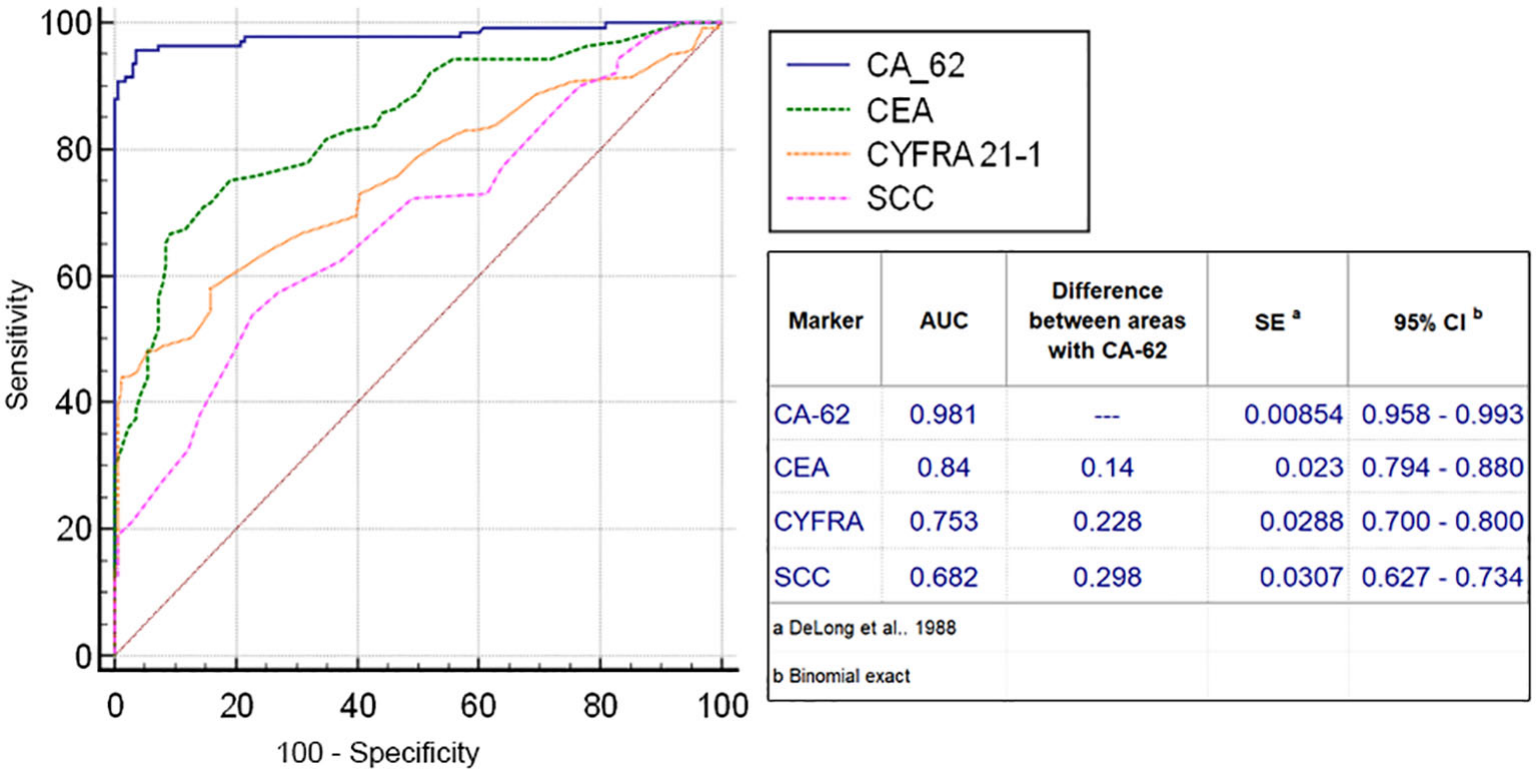
Comparison of ROC curves of CEA, CYFRA 21-1, SCC and CA-62 for all stages of NSCLC.

**Figure 4:**
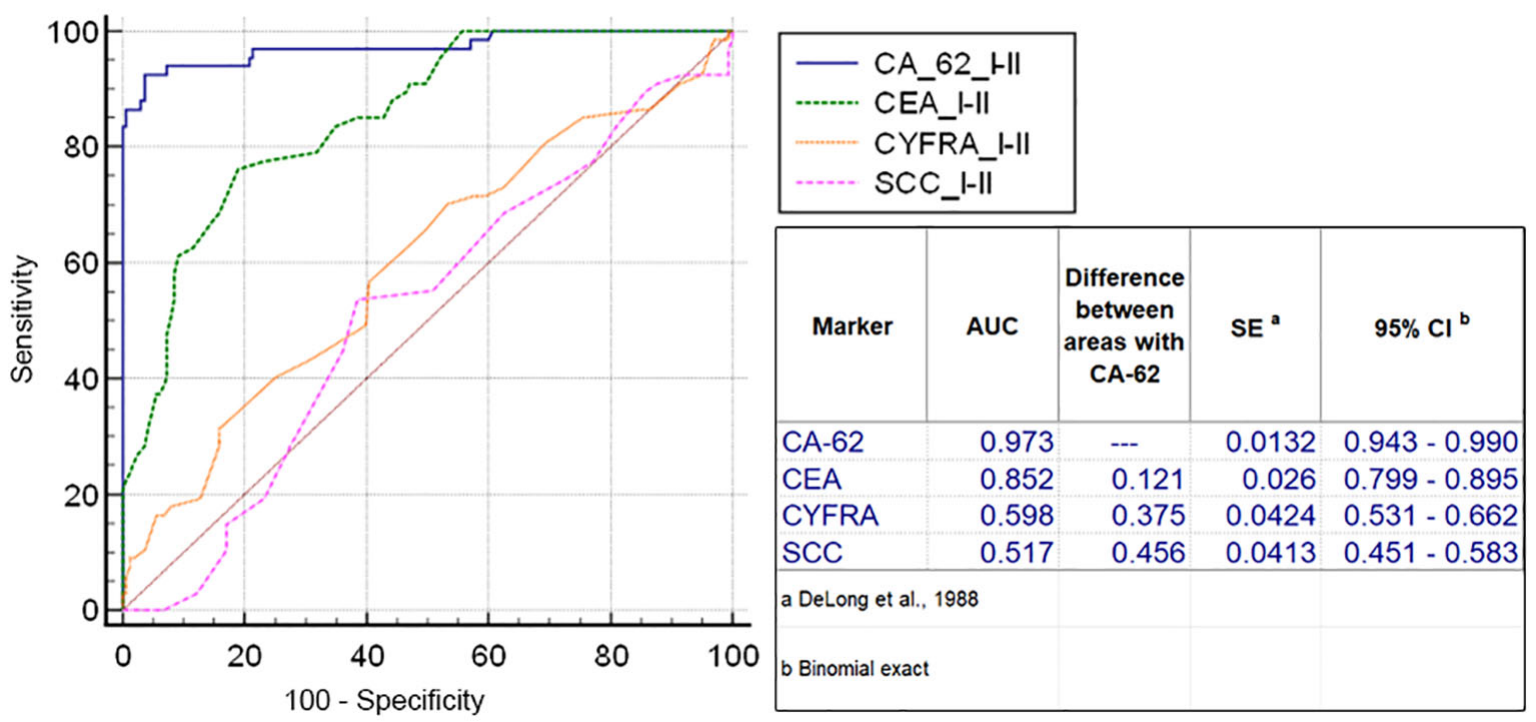
Comparison of ROC curves for CEA, CYFRA 21-1, SCC and CA-62 for early stages of NSCLC.

The diagnostic ROC curves were generated according to the histological TNM classification using a graphical application of MedCalc statistical software (Belgium, EU). Comparison of ROC curves for the most prospective tumor markers, such as CEA, CYFRA 21-1, SCC and CA-62 for the entire NSCLC patient’s cohort against all healthy controls and COPD patients (**Figure 3**) showed a valid difference in the areas under the curve between CA-62 (AUC =0.981) and other TM (area difference AUC = 0.21 - 0.7). The areas under the diagnostic ROC curves were lower and rather expected for other tumor markers: CEA (AUC=0.84) > CYFRA 21-1 (AUC=0.753) > SCC (AUC=0.682). The ability to differentiate NSCLC from a cohort of COPD patients and healthy controls was more significant for CA-62 marker (AUC =0.981) with a diagnostic sensitivity of 95% at 97% specificity (**Figure 3**). **Figure 4** shows the best sensitivity and specificity for detecting early-stage NSCL for CA-62 and CEA markers, with top highest AUC=0.973 for CA-62 and AUC=0.852 for CEA, correspondingly. The CYFRA 21–1 and SCC tumor markers demonstrated insignificant AUC=0.598 and AUC=0.517, respectively.

**Figure 5:**
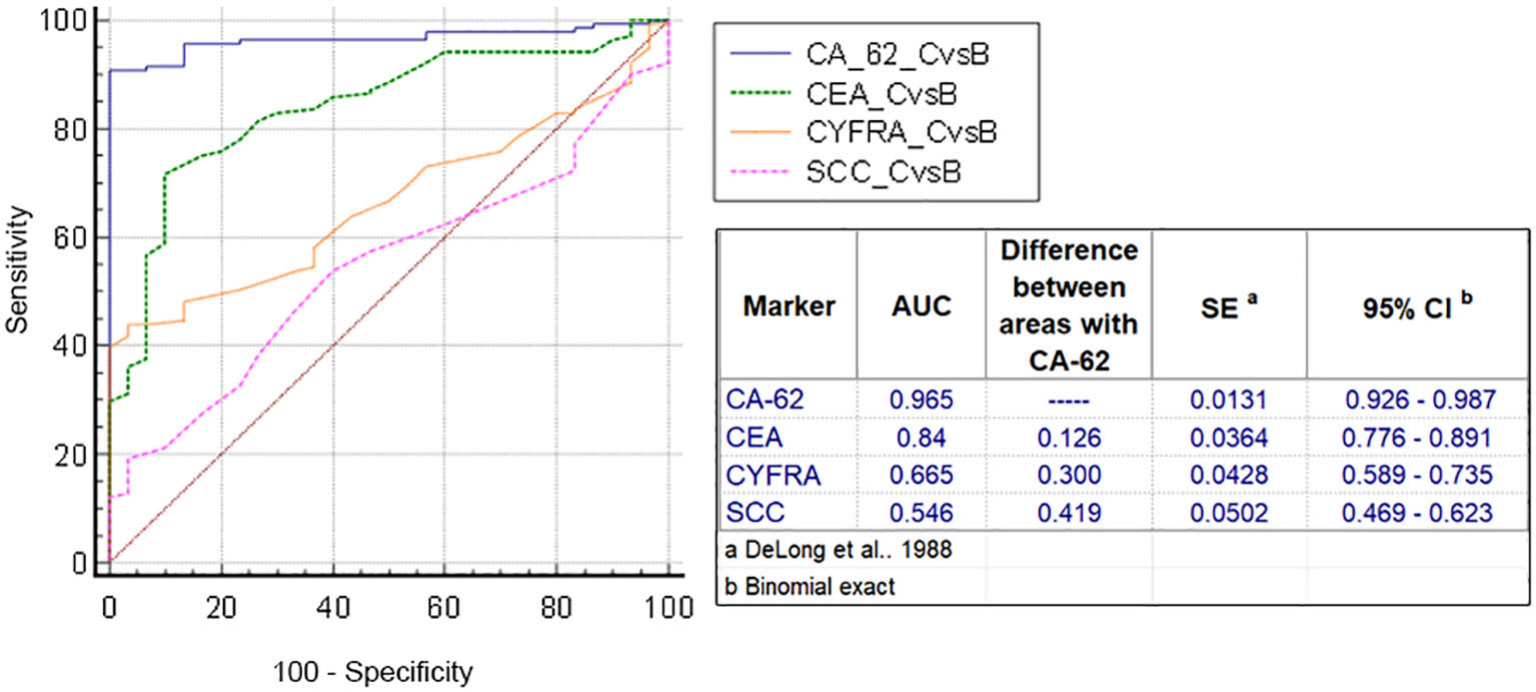
Comparison of ROC curves for markers for NSCLC patients versus cohort of COPD patients.

Using an elevated CA-62 marker cutoff value of 5600 U/ml provides a lowest number of false positives, a high level of specificity (100%) that is required for clinical detection of early stages of lung cancer at screening of patients at risk (heavy smokers over 50). It was derived empirically from the diagnostic ROC curve of NSCLC patients versus cohort of COPD patients (**Figure 5**). A comparison of the AUC difference of ROC curves for CA-62 and CEA confirmed that the CA-62 marker is the most accurate in identifying early stages of NSCLC in asymptomatic patients. Other tumor markers studied, such as CA-125, CA 15-3, CA 19-9, NSE did not have sufficient sensitivity and specificity to diagnose early stages of lung cancer in asymptomatic patients. However, a combination of various biomarkers allows achieving a thorough discrimination between benign and malignant neoplasms better than any single TM.

### 3.3 Combination of tumor markers for early-staged NSCLC detection

There are a few mathematical methods to combine TM. The first method is rather simple and based on averaging of the values for all 3 TM after their normalization. For normalizing all the markers, we used one reference cutoff value of the selected TM (CEA, cutoff 5 ng/ml). In this case, all sample values for CYFRA 21–1 were multiplied by 2 and divided by a 1000 for CA-62 marker.

To plot the ROC curve for the combination of CA-62, CEA and CYFRA 21–1 tumor markers using defined parameters were implemented the following mathematical techniques:

1) A conversion of three independent parameters, such as CA-62, CEA and CYFRA 21–1 values, to one arbitrary value corresponding to the principles-based developed testing approach in accordance with the following mathematical transformations:

(a) Samples were assigned the values equal to:


$$3\;TM=\frac{\sum^{}(\text{CEA}+\left(\frac{\text{CA}62}{1000}\right)+\left(2\times \text{CYFRA}21\_1\right)}{3}[\text{U/ml}]$$

Therefore, samples were considered as “positive” if: 3TM Value > 5 U/ml, and “negative” otherwise. This approach allows the samples to have only one parameter to be considered. The ROC curves were plotted in accordance with the TNM classification. Diagnostic characteristics of CEA CA-62, CYFRA 21-1, and a combination of 3TM (CA-62, CEA and CYFRA 21-1) were evaluated based on their sensitivity and specificity, test accuracy, and curves were compared for different IVD methods. The level of significance was set at P<0.001.

**Figure 6:**
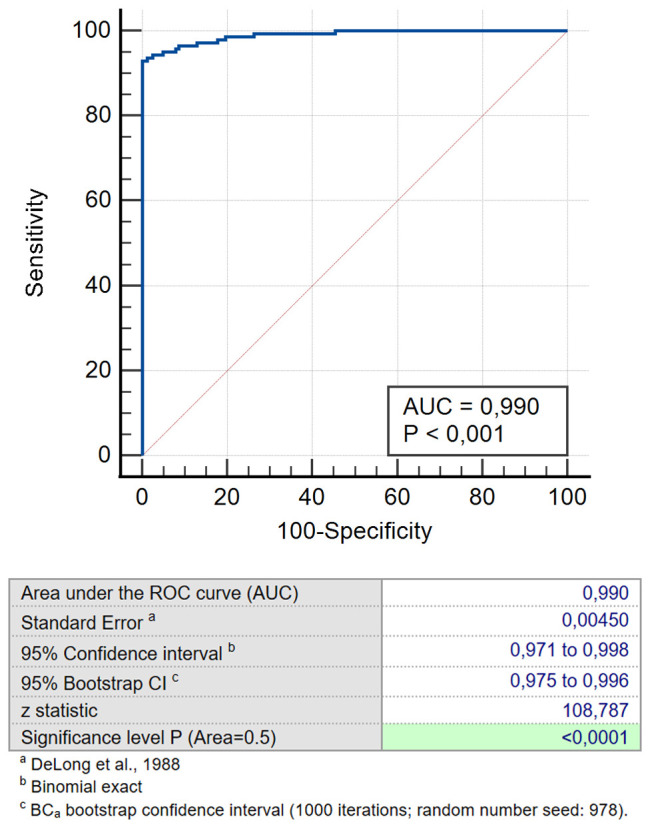
ROC for the predicted probability for combination CEA/CA-62/CYFRA 21-1 for NSCLC patients versus cohort of COPD and healthy patients.

The second method is a linear regression analysis, which is measured as the probability of a binary outcome (e.g. disease or no disease) given the variables used (25). It allows calculating the probability of cancer in a particular patient. For example, in a patient 62 years of age, with CYFRA 21-1 1ng/ml, CEA 4.2 ng/ml, CA-62–6200 U/ml and a positive LDCT scan the probability of having lung cancer is 72%. This is very important for both the doctor and a patient because there is no other way to assess the relative weight of all the variables. A regression model can compare the predicted results with the actual condition, which allows also generating a ROC curve (**Figure 6**). The only difference is that the specificity and sensitivity stem from the combination of all the variables used in the model. Such mathematical model can also assess the significance of each variable used. For example, in combining cancer markers, the value of the markers is highly significant (very low p) whereas age might be not that important (p > 0.05), in which case, we can exclude age as a variable.

We studied a number of various TM combinations (CEA, CYFRA 21-1 & SCC; CEA, CA-125 & CYFRA 21-1; CEA, CA -125 & CA 15-3; CEA, CA-62, CYFRA 21-1 & SCC; CEA,CA-62,CYFRA 21-1 &CA-125; CA 15-3, CA-62 &CYFRA 21-1; CEA, CA-62 and CYFRA 21-1) aiming to find the utmost effective TM panel for early-stage LC detection. Obtained results confirm that a combination of biomarkers selected produces better results for Stage I & Stage II detection than CA-62 cancer marker alone (AUC 0.990 vs 0.973) and other previously proposed panel of markers containing PENK, pro-SP, hGH and CA15-3 (AUC = 0.785) (17). Therefore, adding a cytokeratin-19 fragment CYFRA 21–1 to a combination panel of CA-62 and CEA allows increasing specificity by eliminating false positive results of COPD patients. That improves the diagnostic value of the selected TM panel (CA-62, CEA and CYFRA 21-1) (**Table 3**). The results obtained demonstrated that a combination of two different N- and O-glycoproteins with a 19-citrate fragment (CEA, CA-62 and CYFRA 21-1) allows achieving 100% specificity, 93% sensitivity, and 94% test accuracy for either mathematical method.

**Table 3:** Comparison of diagnostic characteristics of various TMs and their panels used for NSCLC detection.

Marker or marker panel	N patients	Sen %	Sp %	Test accuracy %
CEA, CYFRA 21-1 and SCC (20)	802 LC (472 NSCLC)	82	92	67,5%
СЕА and CYFRA 21-1 (13)	892 patients 655 NSCLC	33	95	49%
СА 125, СA 19-9, СА 15-3 and TAG-72.3 (14)	802 LC 417 NSCLC	81,6%	93,3%	83,6% (Stage I –III)
СЕА, СА 15-3, SCC, CYFRA 21-1, NSE and ProGRP (15)	3144 LC	88,5%	82%	85,8%
	908 NSCLC	87,1%	82%	85,5%
CEA, CYFRA 21-1, SCC, NSE, ProGRP, and CA-125 (15)	2097 patients 1048 NSCLC	87,12	64,6%	83,8%
CEA and OPN (30)	200 patients 80 NSCLC	87,5%	86,7%	86%
СЕА and DKK1 (30)		92,5	76,7%	83%
Autoantibody test EarlyCDT®-Lung (p53, NY-ESO-1, CAGE, GBU4-5, Annexin I, and SOX2) (31)	776	40%	82%	80%
miRNA signature (32)	225	87%	81%	80%
СА-62 (23)	304	96%	97%	96,7%
CEA, СА-62 and CYFRA 21-1 (23)	304	93%	100%	94%

A comparative analysis of the data presented on various TM and their panels used in lung cancer diagnosis worldwide showed that the CA-62 marker and its panel (CA-62, CEA and CYFRA 21-1) has the highest diagnostics characteristics as compared to other laboratory diagnostic methods for estimating lung malignancies currently available.

## 4 Discussion

In this article we evaluated the diagnostic characteristics of the CLIA-CA-62 set of reagents for the detection of early (I-II) and advanced stages of lung cancer in patients with pathological changes on the CT scan compared to other tumor markers (CEA, CA-125, CA15-3, SCC, CYFRA 21-1, NSE and CA 9-19) on a cohort of 304 patients.

Lately the most effective method of LC screening has been the LDCT, which allows the detection of 50-72% early stages as compared to lung cancer detection by presentation (20-29%) (1, 2). The results of the National Lung Screening Trial (NLST) conducted in Italy in 2011, which included three consecutive LDCT studies of more than 53,000 people (26, 27) showed a 20% reduction in LC mortality and a 7% overall mortality reduction. The results of the screening program determined the necessary number of LDCT studies of asymptomatic patients in the high-risk group to identify one verified case of LC (36) and one case of LC in the early (I-II) stages (90) for two years of screening (27). As a result of LDCT screening, the ratio of early stages (I - II) of LC increased by 37.5%: from 28.5% to 52% after first LDCT scan and 66% after second CT-scan. According to the international randomized clinical studies LDCT screening showed its effectiveness as a method in reducing the mortality from LC by 23% in the first year of screening. Without screening, mortality from LC was 525 and with screening it decreased to 295. However, preventing one person from dying from LC requires 320 LDCT studies (28).

Despite the obvious advantages of LDCT screening, it has serious drawbacks. The authors (28) estimated the prevalence of pre-clinical LDCT and the positive predictive value of LDCT diagnostics, which turned out to be quite low (< 20%) for all certain target groups of heavy smokers according to age and smoking history. Global hyper diagnosis of LC in screening is also a serious problem, estimated by various studies to be between 18% and 67% (5, 6, 29). In the many countries in the world, the LDCT method does not allow its wide use in organized population screening due to the limited availability and capacity of LDCT scanners. The availability of low-dose CT scanners is particularly problematic in remote and rural areas worldwide.

It is important to clarify several points regarding the practical integration of CA-62 testing prior to LDCT (low-dose computed tomography):

### 4.1 Feasibility and clinical infrastructure

LDCT remains resource-intensive, requiring specialized equipment, trained technicians, radiologists, and physical infrastructure. Its implementation is often limited by availability and scheduling bottlenecks. In contrast, CA-62 testing is based on a simple blood draw, compatible with existing venous sampling workflows, including mobile and remote settings.

### 4.2 Cost and scalability

The CA-62 immunoassay, while novel, is built on established immunoassay platforms and does not require costly imaging or capital-intensive infrastructure. Unlike LDCT, which incurs fixed high costs per scan, the cost of CA-62 testing decreases significantly with scale, due to relatively stable reagent use and automation potential. We estimate that the direct cost per test can be reduced to below USD 20 in high-throughput settings, which is comparable to other standard IVD immunoassays (PSA, CEA).

### 4.3 Minimizing false positives

While false positives are a known concern in screening, our modeling shows that incorporating CA-62 as a pre-screening filter reduces the number of patients requiring LDCT by a factor of 15–16 times, based on calculated PPV from real-world prevalence, sensitivity, and specificity.

In addition to LDCT, the use of tumor markers for monitoring the treatment of LC patients and monitoring their condition after reaching remission has been widely implemented as for evaluating the effectiveness of chemotherapy performed, and for the detection of relapse and dynamic surveillance of patients with LC (8, 13–16). Examples of such markers include CEA, NSE, SCC, CYFRA-21, and CA-125, but all these individual biomarkers are not sensitive enough to identify a reliable direct relationship between disease progression and their elevation. The limitation on TM use for LC detection is due to their low sensitivity (15-40%) at detecting early stages of cancer (21, 22).

The results of the blind clinical study described in this article showed the most significant diagnostic characteristics using the carcinoma-specific marker CA-62 for the detection of NSCLC (stages I-III): 96% sensitivity at 97% specificity, compared to other tumor markers studied by us or their combinations described in the literature (**Table 3**). The best sensitivity values for early-stage detection (I and II) NSCLC were observed for CA-62 (92%) among all TMs: CEA (37%), CYFRA 21-1 (9%), SCC (9%), NSE (4.5%), CA-125 (3%), CA 15-3 (1.5%), and CA 19-9 (1%) (**Figure 1**). The increased CA-62 marker cutoff value (5,600 U/ml) allowed the elimination of false positives, reaching high specificity (100%) at 89% sensitivity required for clinical detection of early-stage lung cancer.

An additional evaluation of the marker application efficacy includes the calculation of the weighted kappa coefficient (k), which is clearly demonstrates the difference between various diagnostic methods, exactly which biomarker or their combination classifies patients more reliably due to the lesser likelihood of a random coincidence of the test results with the histopathological findings (17). The exact values of the weighed kappa-coefficient demonstrate a significant difference between two diagnostic methods: an agreement between the two estimation methods is considered bad if 0< k< 0.20 and good if k > 0.81. When evaluating the detection effectiveness of NSCLC patients from the entire cohort, the values of kappa coefficients were distributed as follows: CA-62 (k=0.92) > CYFRA 21-1 (k=0.44) > CEA (k=0.43) > SCC (k=0.19). Similar results were obtained for the evaluation of the similar criteria in the cohort of patients with early stages of NSCLC: CA-62 (k=0.88) > CYFRA 21-1 (k=0.09) > CEA (k= 0.37) > SCC (k= 0.09). Therefore, from the entire panel of tumor markers CA-62 is the only marker that demonstrated high correlation with histopathology results in terms of recognition of malignant processes in nodules found incidentally on LDCT-scan or in the presence of pathological pulmonary changes, including infiltrates.

It is important to note that unlike mucins and other tumor markers, which are increased proportionally to the tumor growth, the marker CA-62 is significantly elevated from Stage I and demonstrated the highest diagnostic characteristics in detecting early stages (I & II) of NSCLC (Sen=91- 96.4%) (**Figure 2**).

This is undoubtedly that CA-62 has a great advantage over other tumor markers with much lower sensitivity (7.1% – 48.2%) for detecting early stages of lung cancer. In particular, the biomarkers CYFRA 21-1 (Sen=44%), SCC (Sen=18.4%), and CA 19-9 (Sen=7.1%) do not have sufficient sensitivity to diagnose asymptomatic lung cancer, however at the same time they have high enough specificity to differentiate between the benign and malignant lung neoplasms.

Another approach for eliminating false positive results consists in combining highly sensitive cancer antigen CA-62 with other biomarkers. Therefore, an addition of cytokeratin 19 CYFRA 21–1 to glycoproteins CA-62 and CEA increases the specificity of early-staged NSCLC detection by eliminating false positive results, which significantly improves the diagnostic value of the tumor marker signature (CA-62, CEA & CYFRA 21-1): 100% Specificity, 93% Sensitivity and 94% test accuracy (**Table 3**), highlighting the added value of CA-62 as a complementary marker that enhances both early detection and diagnostic precision. Using various mathematical approaches (using an average of biomarkers combination or regression analysis) allows achieving high diagnostic characteristics of chosen expanded TM combination (CEA, CA-62 & CYFRA 21-1) and getting a probability estimation of having LC using regression.

The results of the clinical study demonstrated that using the biomarkers signature (CA-62, CEA and CYFRA 21-1) allows increasing the Specificity of CT diagnostics for patients with suspicious changes on the tomogram, improving the interpretation of visualized localized focus, and improving the accuracy of differential diagnosis at detecting early stages of LC up to 94%. These findings suggest that CA-62 not only confirms the prior utility of CEA and CYFRA 21–1 but also extends their effectiveness into earlier disease stages, offering new opportunities for pre-symptomatic identification of high-risk individuals.

The unique feature of highly glycosylated N-glycoprotein CA-62 consists in its expression in large concentration on the cell membrane of transformed maligned stem cells from the onset of carcinogenesis, long before any clinical symptoms appearance (10, 11). The Sensitivity, Specificity and CLIA-CA-62 Test accuracy for the entire cohort of NSCLC patients were high at 96%, 97%, and 96.7%, which is higher than the sum of other tumor markers and their panels presented in **Table 3**, which are currently used in LC diagnosis. Other lung cancer diagnostic methods have either a low sensitivity of 25-40% to 80%, depending on the stage of disease, or low specificity values. The Molina R. et al. (14) group’s studies have demonstrated that successful TM combinations have enough diagnostic sensitivity to detect LC. Clinical studies conducted by the authors on 802 patients demonstrated that using of a combination of several known markers (CEA, CYFRA 21–1 and SCC) for NSCLC and (ProGRP, NSE, CEA or CYFRA 21-1) for small-cell lung cancer (SCLC) allow reaching Sen=80% in LC patients with Sp = 92% at Stages I-III. Studies by Kyoko Okamura et all (13) confirmed the high diagnostic value of CEA and CYFRA 21–1 tumor markers for differentiating 655 patients with primary LC from 237 COPD patients. The sensitivity and specificity of CEA for LC detection was 69% and 68%, respectively, compared to 43% for CYFRA 21–1 at Sp=89%. The combined use of these two markers revealed a more significant result compared to individual TM at Sen=33% and Sp=95% for LC with a prevalence rate of 51% of patients at risk. Later studies of the Molina et al. (15) identified six serum TM (CEA, CA 15-3, SCC, CYFRA 21-1, NSE and progastrin releasing peptide ProGRP) that were associated with the histological subtypes of LC: NSCLC and SCLC. The authors showed that combined use of the 6 tumor markers described above showed better diagnostic characteristics for NSCLC detection as compared to the individual characteristics of tumor markers: better sensitivity, specificity PPV and NPV (87.1%, 82%, 85.3% and 84.3%, respectively. Jinlin Sun et al. (30) investigated another interesting combination of TM for NSCLC detection: CEA, secretory sialoprotein osteopontine (OPN) and secreted protein Dickkopf-1 (DKK1), which showed promising results. The sensitivity and specificity of the (CEA and OPN) combination for NSCLC (AUC=0.920) (95% CI, 0.875-0.964) were 87.5% and 86.67%, respectively. The area under the ROC curve of CEA and DKK1 for NSCLC was AUC=0.912 (95% CI, 0.866-0.958) with sensitivity and specificity 92.5% and 76.67%, respectively. It is important to clarify that the direct comparisons of different methods used are limited due to population heterogeneity, different assays, and statistical handling.

Over the past l0 years, a significant amount of research data has accumulated on other promising biomarkers for lung cancer detection: autoimmune antibody signature (Autoantibody signature, AAB) (31), micro-RNA (32) and pro-surfactant Protein B (Pro-SFTPB) (33). The presence of such biomarkers in plasma or serum of patients could be an independent predictor of lung cancer and may be a valuable addition to existing lung cancer screening models.

Finally, the results obtained confirm that the combined use of TM, in particular CEA, CA-62 and CYFRA 21–1 could be a useful strategy for improving of the integrated LC risk evaluation in a high-risk group. Reducing the proportion of false positives in initial LDCT scans, as well as reducing LC hyper diagnosis using a more effective evaluation of tumor aggressiveness, is an important and as yet unachieved clinical challenge.

## 5 Conclusions

The use of CA-62 biomarker alone or in combination with CEA and CYFRA 21–1 can be as effective methods of lung cancer pre-screening tool before or after LDCT scans or after it as a marker of decision on surgical biopsy was demonstrated by the results obtained in a blind clinical study. As a result of the conducted retrospective double-blind study, we can provide approaches to develop protocols for future prospective of such clinical studies. The results obtained showed high diagnostic characteristics of detecting early (I & II) stages of lung cancer and can be used via diagnostic decision. The existing LDCT screening of LC can be significantly improved by sequential use of CT- scan as a diagnostic tool for visual detection of lung nodules and *in vitro* diagnostic CLIA-CA-62 immunoassay.

Our findings indicate that the combination of CA-62, CEA, and CYFRA 21–1 is a reliable TM panel for early-stage lung cancer detection, in addition to CT scans in high-risk groups. The conclusions of a double-blind clinical study were:

1. Unlike other tumor markers, such as CEA, CA 15-3, CA-125, NSE, CA 19-9, CYFRA 21-1, and SCC, which are elevated respectively to the tumor growth, the use of highly sensitive CA-62 for detection early stages of lung cancer (Ia - IIb) demonstrated the ultimate diagnostic characteristics: 92.5% Sensitivity, 96.3% Specificity and 95% accuracy of LC detection at biopsy.
2. A comparison of various tumor markers results obtained for the serum samples of the entire cohort of 141 NSCC patients (I-III) has reliably shown that the level of cancer antigen CA-62 is significantly increased in 136/141 (96.4%) NSCLC patients with 97% Specificity compared to other tumor markers: 48.2% for CEA, 44% for CYFRA 21-1, 22% for CA-125, 19% for CA 15-3, 18.4% for SCC, 10% for CA 19–9 and 9.2% for NSE, which often «miss» early stages of lung cancer.
3. The results of the clinical study demonstrated that using the biomarkers combination (CA-62, CEA and CYFRA 21-1) allows increasing the Specificity of CT diagnostics for patients with pathological changes on the tomogram, improving the interpretation of visualized localized focus, and improving the accuracy of differential diagnosis at detecting early stages of LC up to 94%. This can accelerate timely treatment and improve overall patient survival.
4. In the prospective, adding cancer marker CA-62 alone or in a panel TM signature (CA-62, CEA and CYFRA 21-1) to the existing LC risk assessment system as a pre-screening tool for LDCT-scan may improve the quality of early-stage lung cancer detection by significantly increasing the sensitivity and by reducing the proportion of false positive results. The number of patients who require LDCT scan can be significantly reduced, which can allow this screening algorithm to fully fit into the throughput of Low-Dose CT lung cancer organized screening and reduce the radiation load on the body and overcome other barriers of LDC.
5. Overall, the introduction of CA-62 or a panel (CA-62, CEA and CYFRA 21-1) screening into the health system prior to LDCT lung cancer screening can reduce the overall cost of LC screening programs by dropping the burden of LDCT and conducting additional testing for patients with suspicious tomograms. The lack of general access to LDCT screening in remote and rural regions is particularly evident.

To reliably detect early stages of lung cancer, it is essential to evaluate the effectiveness of the TM panel (CA-62, CEA and CYFRA 21-1) or CA-62 in specific clinical applications, such as a pilot screening program for patients in high-risk group (age over 50, chain smokers). Such clinical studies have the potential to provide insight into the usefulness of the CA-62 biomarker or the TM panel (CA-62, CEA and CYFRA 21-1) as a first-line testing for selecting subjects in the high-risk group of LC development who require further LDCT screening, potentially avoiding radiological exposure of people of low-risk of LC with negative scans.

To develop a working algorithm for differential diagnosis of pulmonary nodules using the TM panel (CA-62, CEA and CYFRA 21-1), it is also useful to conduct a prospective clinical study on a group of patients with pathological changes in the lungs on a CT scans. The decrease in the ratio of false positive results obtained in the sequential use of LDCT scan and the TM panel (CA-62, CEA and CYFRA 21-1) is of great clinical importance in the context of improving Low-Dose CT lung cancer organized screening and reducing potential side effects, associated with repeated CT scans or other unnecessary invasive diagnostic methods.

In summary, we believe that incorporating CA-62 prior to LDCT could offer a cost-effective, scalable, and logistically feasible triage tool, particularly valuable in settings where imaging resources are constrained.

## Data Availability

The raw data supporting the conclusions of this article will be made available by the authors, without undue reservation.
